# Knockout of Ccr2 alleviates photoreceptor cell death in rodent retina exposed to chronic blue light

**DOI:** 10.1038/cddis.2016.363

**Published:** 2016-11-10

**Authors:** Zizhong Hu, Yi Zhang, Junling Wang, Pingan Mao, Xuehua Lv, Songtao Yuan, Zhengru Huang, Yuzhi Ding, Ping Xie, Qinghuai Liu

**Affiliations:** 1 Department of Ophthalmology, The First Affiliated Hospital of Nanjing Medical University, Nanjing 210029, China

## Abstract

Age-related macular degeneration (AMD), the leading cause of visual loss after the age of 60 years, is a degenerative retinal disease involving a variety of environmental and hereditary factors. Although it has been implicated that immune system is involved in the disease progression, the exact role that microglia has is still unclear. Here we demonstrated that knockout of *Ccr2* gene could alleviate photoreceptor cell death in mice retinas exposed to chronic blue light. In *Ccr2*^**−/−**^ mice, a damaged microglia recruitment was shown in retina and this could protect the visual function in electroretinogram and alleviate the photoreceptor apoptosis, which thus helped attenuate the blue light-induced retinopathy. We further found an increased co-location of NLRP3, Iba-1, and IL-1*β* in fluorescence and a concomitant increased protein expression of NLRP3, caspase-1, and IL-1*β* in western blotting in chronic blue light-induced retinopathy. Moreover, the activation of microglia and their cellular NLRP3 inflammasomes occurred as an earlier step before the structural and functional damage of the mice retinas, which collectively supported that microglial NLRP3 inflammasome might be the key to the chronic blue light-induced retinopathy.

Photoreceptor cell death is irreversible in retinal diseases and can cause the loss of central vision, night blindness, and constriction of the visual field. In age-related macular degeneration (AMD), the leading cause of vision loss among the elderly worldwide,^1, 2^ apoptosis of photoreceptor cells is known as the final common pathway even though its exact etiopathogenesis is poorly understood.^3^ Thus it is of great importance to investigate how risk factors initiate the early retinal damage and to develop therapeutic strategies to prevent its progression.

Epidemiological studies have suggested an association between visible light exposure and AMD risk.^4, 5^ Light-induced retinopathy has also been studied *in vivo* for over 50 years as a model for human retinal degenerative diseases.^6, 7, 8, 9^ Severity of the light-induced retinopathy depends mainly on wavelength, intensity, and exposure time of illumination source. Extensive work of animal models of light-induced retinopathy, however, mostly have applied acute high-intensity light.^10, 11, 12^ Though exposure to high-intensity light can induce photo-oxidative stress and some features of atrophic AMD,^13^ this model cannot perfectly mimic the human retinopathy, especially the early retinal alteration in consideration that light-induced retinopathy in AMD is actually a long-term process. Thus a low-intensity, long-term light exposure is called for the study of pathogenesis of the light-induced retinopathy.^14, 15^

Growing evidence has suggested that inflammation, immune response, and genetics may have important and interacting roles in AMD.^16, 17, 18, 19^ During the past decades, the pathogenesis of neurodegenerative diseases in central nervous system (CNS) has shed light to AMD research. First, microglia, the resident macrophages of the CNS, which originate from circulating bone marrow-derived monocytes, are key players in both acute and chronic inflammatory processes.^20, 21, 22^ In AMD patients, microglia are activated and accumulate in the subretinal space.^23, 24^ Of note, microglia have a controversial role in light-induced retinopathy. Although others suggested that microglia could remove apoptotic photoreceptors and cell debris in the injured retina and benefit photoreceptor survival,^25, 26^ our work^27, 28^ and other groups^29, 30^ indicated excessive recruitment and activation of microglia could precede photoreceptor degeneration and was associated with proinflammatory and neurotoxic cytokines in the affected regions. A noteworthy fact is that no study has so far focused on the controversial role of microglia in chronic light-induced retinopathy. Second, studies on CNS disease have focused on NLR family pyrin domain containing 3 (NLRP3) inflammasome activation. The NLRP3 inflammasome is a multiprotein complex that recruits caspase-1 and mediates the production of interleukin-1 beta (IL-1*β)* in microglia.^31, 32^ More recently, in AMD pathogenesis, NLRP3 inflammasome was reported mainly activated in retinal pigment epithelium (RPE) cells.^33, 34, 35, 36^ However, important questions remain, such as whether inflammasome activation is involved in microglia in retina under chronic light exposure, whether this activation leads to light-induced retinopathy, and can the retinopathy be alleviated through impairment of microglia recruitment.

To elucidate the effect of microglia in chronic light-induced retinopathy, we performed our experiment on *Ccr2*^**−/−**^ mice with a focus on NLRP3 inflammasome activation in microglia. This study further supports our hypothesis that microglial NLRP3 inflammasome activation may be a mediator of IL-1*β*-related photoreceptor degeneration in light-induced retinopathy.

## Results

### C-C motif chemokine receptor-2 (Ccr2) knockout protects vision function in electroretinogram (ERG)

**Figure 1:**
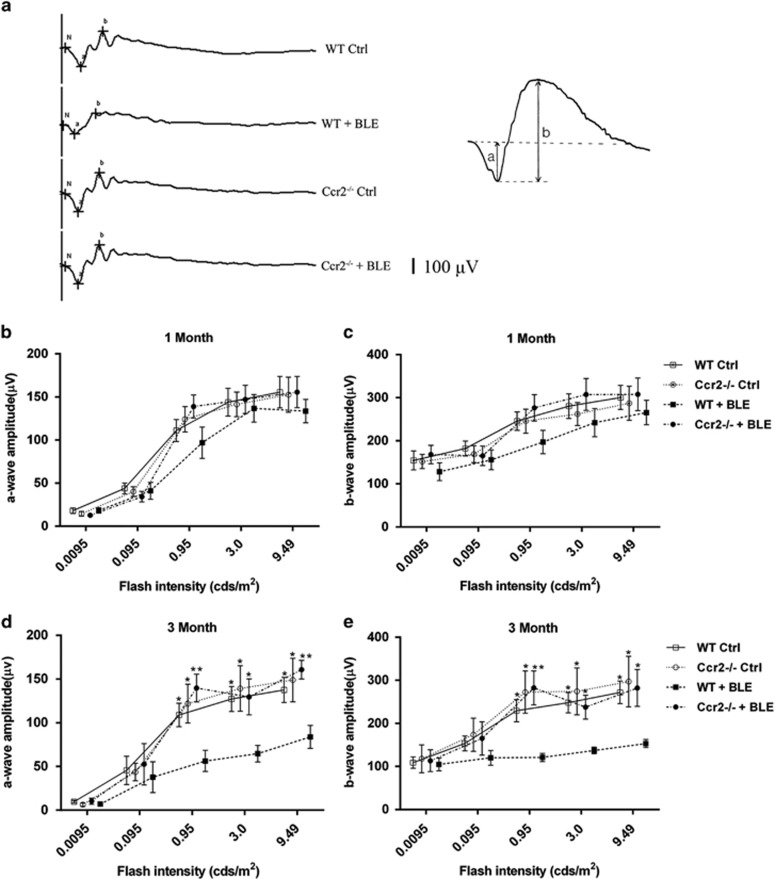
Effect of *Ccr2* knockout on light-induced retinal dysfunction in mice. Stimulus flash intensity was selected from 0.0095 to 9.49 cds/m^2^ in ERG analysis. (**a**) Representative scotopic ERG records at the flash intensity of 3.00 cds/m^2^ after 3 months in the four groups. Quantification of (**b**) a-wave amplitude and (**c**) b-wave amplitude of *WT Ctrl*, *Ccr2*^**−/−**^ *Ctrl*, *WT+BLE*, and *Ccr2*^**−/−**^*+BLE* mice after 1 month. Quantification of (**d**) a-wave amplitude and (**e**) b-wave amplitude of *WT Ctrl*, *Ccr2*^**−/−**^ *Ctrl*, *WT+BLE*, and *Ccr2*^**−/−**^*+BLE* mice after 3 months. Data are shown as the mean±S.E.M., *n*=10. One-way analysis of variance followed by Tukey's multiple comparison test. *P<0.05, **P<0.01, *WT+BLE* group compared with the other three groups

Flash ERGs were recorded 1 month and 3 months after light exposure in the four experimental groups. Both a- and b-wave amplitudes were slightly reduced in the wild-type+blue light exposure (*WT+BLE*) group compared with the *WT Ctrl* group after 1 month. The reduction, however, was not statistically significant (Figures 1b and c). If we were to increase the exposure duration to 3 months, a remarkable decrease in a- and b-wave amplitudes were then caused in the *WT+BLE* group (Figures 1a, d, and e). Take the flash intensity of 3.00 cds/m^2^ for instance, the amplitudes of a- and b-wave in the *WT+BLE* groups decreased by 49.6% (*P*=0.011) and 44.7% (*P*=0.022), respectively, compared with the control (Ctrl) groups. Statistical difference also existed when the flash stimulus of 0.95 and 9.49 cds/m^2^ were applied. However, knockout of *Ccr2* prevented the reduction of a- and b-wave amplitudes in the *Ccr2+BLE* group (Figures 1a, d, and e). The a- and b-wave responses, after 1 month and 3 months, were not significantly different among the three groups of *WT Ctrl*, *Ccr2*^**−/−**^ *Ctrl*, and *Ccr2*^**−/−**^*+BLE* at all flash intensities (Figures 1b–d).

### Ccr2 knockout prevents ONL thinning in histological analysis

**Figure 2:**
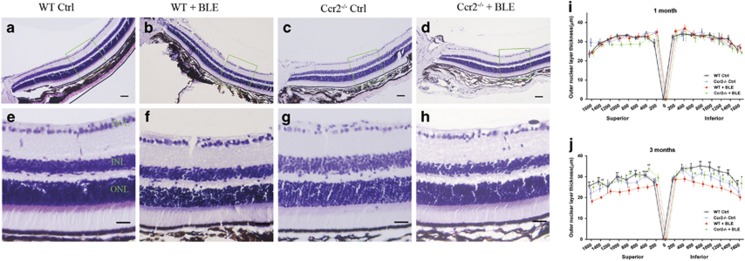
Effect of *Ccr2* knockout on ONL thinning induced by exposure to chronic blue light in mice. Representative retinal images of H&E staining in the (**a** and **e**) normal *WT Ctrl* mice, (**b** and **f**) *WT+BLE* mice (500 lux), (**c** and **g**) *Ccr2*^**−/−**^ *Ctrl* mice, and (**d** and **h**) *Ccr2*^**−/−**^*+BLE* mice (500 lux). Panels (**a**–**d**) are retinal segments between optic nerve and approximately 1000 *μ*m from the optic nerve with a scale bar of 50 *μ*m. Panels (**e** and **f**) are retinal segments between 400 and 600 *μ*m away from optic nerve in the superior area with a scale bar of 20 *μ*m (green box in panels (**a**–**d**)). (**i**) Measurement of thickness of the ONL in the four groups after 1 month. (**j**) Measurement of thickness of the ONL in the four groups after 3 months. Data are shown as mean±S.E.M., *n*=6. Significant differences were calculated using one-way analysis of variance followed by Tukey's multiple comparison test. *P<0.05, **P<0.001 compared with *Ccr2*^**−/−**^*+BLE* mice

Histological analysis showed that, after 1 month, the average thickness of outer nuclear layer (ONL) did not change much in *WT+BLE*, *Ccr2*^**−/−**^ *Ctrl*, and *Ccr2*^**−/−**^*+BLE* mice compared with the normal *WT Ctrl* mice (Figure 2i). After 3 months of BLE, the ONL thickness was remarkably reduced in the *WT+BLE* group (Figure 2j). Figures 2a–h show the representative retinal hematoxylin and eosin (H&E)-staining images between the optic nerve and 1000 *μ*m from the optic nerve in the superior area after 3 months in the four groups. No reduction was induced by *Ccr2* knockout when comparing *WT Ctrl* mice with *Ccr2*^**−/−**^ *Ctrl* mice (*P*>0.05 for each retina area). The *Ccr2* knockout, whereas, significantly protected the retina from blue light-induced reduction of ONL thickness (Figures 2d, h, and j).

### Ccr2 knockout alleviates photoreceptor apoptosis

**Figure 3:**
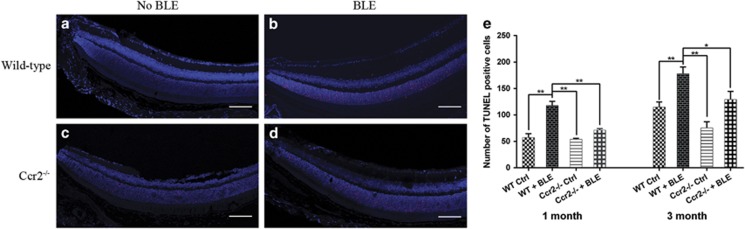
*Ccr2* knockout suppresses blue light-caused photoreceptor apoptosis in the mice retina. TUNEL staining of retinal sections starting from the optic nerve of (**a**) *WT Ctrl* mice and (**c**) *Ccr2*^**−/−**^ *Ctrl* mice. After 3 months of BLE, (**b**) a significant increase of apoptotic photoreceptors was caused in *WT+BLE* mice (**d**) while *Ccr2* knockout significantly suppressed the blue light-induced photoreceptors apoptosis. TUNEL-positive cells (red) mainly expressed in the ONL. Cell nuclei (blue) were counterstained with DAPI. (**e**) Quantitative analysis of TUNEL-positive cells in ONL after 1 month and 3 months in four groups. Data are shown as the mean±S.E.M., *n*=5–6. One-way analysis of variance followed by Tukey's multiple comparison test. *P<0.05, **P<0.001

Terminal deoxynucleotidyl transferase (TdT)-mediated dUTP nick-end labeling (TUNEL) assay showed the number of apoptotic photoreceptor cells in *WT+BLE* mice was remarkably higher than that in normal *WT Ctrl* mice (Figures 3a and b). Knockout of *Ccr2* could suppress the photoreceptor apoptosis (Figure 3d). Further, we quantified the apoptotic cells in the retinas (Figure 3e). After 1 month, the average apoptotic number of either *WT+BLE* mice or *Ccr2*^**−/−**^*+BLE* mice was higher than their corresponding Ctrl groups. In statistic, however, only the number of *WT+BLE* group was significantly higher (117.60±18.26) than that in the *WT Ctrl* (57.25±14.45, *P*<0.001), *Ccr2*^**−/−**^ *Ctrl* (54.00±3.60, *P*<0.001), and *Ccr2*^**−/−**^*+BLE* groups (71.25±5.85, *P*<0.001), whereas no statistical difference was found between the *Ccr2*^**−/−**^ *Ctrl* and *Ccr2*^**−/−**^*+BLE* groups (*P*>0.05). After 3 months, the average apoptotic number of each group increased. Compared with the *WT Ctrl* (114.75±19.80) and *Ccr2*^**−/−**^ *Ctrl* groups (75.50±23.50), the average apoptotic number was significantly higher in the *WT+BLE* group (178.00±28.24, *P*=0.004, *versus WT Ctrl*) and slightly higher in the *Ccr2*^**−/−**^*+BLE* group (129.20±34.31, *P*=0.012, *versus Ccr2*^**−/−**^ *Ctrl*). Of note, the average apoptotic number of the *WT+BLE* group was also significantly higher than that in the *Ccr2*^**−/−**^ *Ctrl* (*P*<0.001) and *Ccr2*^**−/−**^*+BLE* groups (*P*=0.015). These results, along with the flash ERG and ONL thickness analysis, suggested that (1) low-intensity blue light could initially cause the apoptosis of retinal photoreceptor cells, (2) the apoptosis of the photoreceptors was then followed by the reduction of ONL thickness and the impairment of visual function with the extending of BLE, and (3) the *Ccr2* knockout was capable of alleviating the photoreceptor cell death and protecting the visual function of mice after BLE.

### Ccr2 knockout inhibits microglia activation in light-induced retinal degeneration

**Figure 4:**
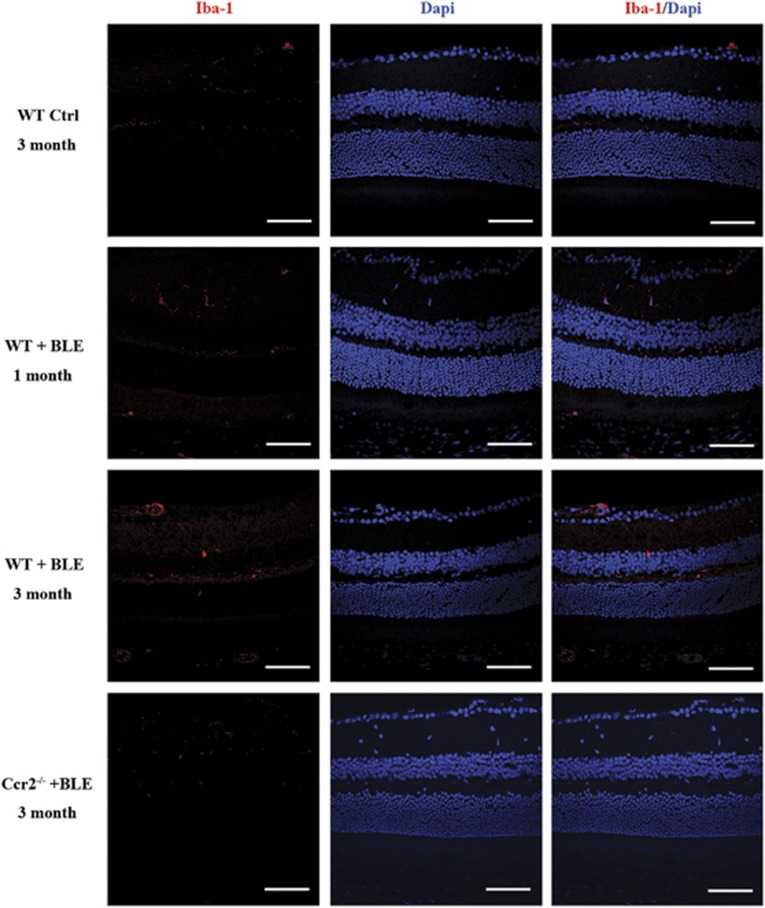
Immunofluorescence of activated microglia in the mice retina. Activated microglia were labeled with an antibody against Iba1 (red) and cell nuclei were labeled with DAPI in the representative retina of *WT Ctrl* after 3 months, *WT+BLE* after 1 month and 3 months, and *Ccr2*^**−/−**^*+BLE* after 3 months. More microglia were activated in the *WT+BLE* mice retina

**Figure 5:**
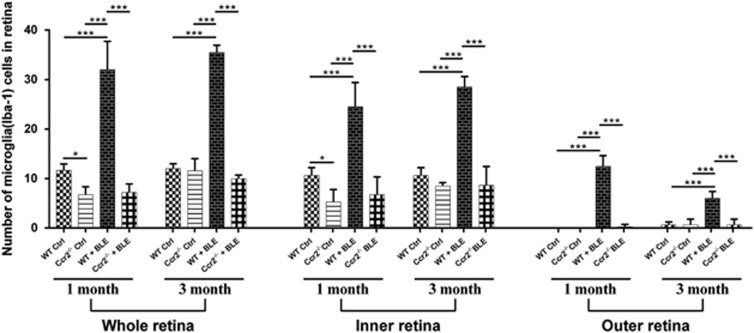
Quantification of activated microglia in the mice retina. After 1 month and 3 months of BLE, the average numbers of microglia in the whole retina, inner retina, and outer retina were significantly higher in the *WT+BLE* mice than that in the *WT Ctrl* and *Ccr2*^**−/−**^*+BLE* groups. Data are shown as the mean±S.E.M., *n*=5–6. One-way analysis of variance followed by Tukey's multiple comparison test. *P<0.05, ***P<0.001

Above results have demonstrated no difference between the *Ccr2*^**−/−**^ *Ctrl* and *WT Ctrl* mice in visual function and retinal morphology, so further experiments focused on the comparisons of *WT Ctrl*, *WT+BLE*, and *Ccr2*^**−/−**^*+BLE* mice. The ionized calcium-binding adaptor molecule 1 (Iba-1) fluorescence identified that *WT+BLE* mice had significantly more activated microglia compared with *WT Ctrl* mice, although in *Ccr2*^**−/−**^*+BLE* mice microglia activation was significantly inhibited (Figure 4). Figure 5 shows the quantification of the activated microglia nuclei in the whole retina, inner retina, and outer retina. When without BLE, the microglia in whole retina was inhibited by *Ccr2* knockout with its quantified number diminishing from 11.66±3.14 to 6.75±3.59 (*P*=0.006) after 1 month. However, this difference between the *WT Ctrl* and *Ccr2*^**−/−**^ *Ctrl* groups disappeared after 3 months. After 1 month of BLE, activated microglia were significantly increased in the *WT+BLE* group with a total number of 32.00±9.89 compared with 11.66±3.14 in the *WT Ctrl* group (*P*<0.001). At the same end point, the number of activated microglia in *Ccr2*^**−/−**^*+BLE* was 7.20±4.20 (*P*<0.001). After 3 months, the quantified number of activated microglia of the *WT+BLE* group increased to 35.50±3.28, which was also significantly higher compared with that of the *WT Ctrl* (12.00±2.24, *P*<*0.001*) and *Ccr2*^**−/−**^*+BLE* (10.00±1.41, *P*<0.001) groups (Detailed quantification: Supplementary Table S1). Subgroup quantification of the number of Iba-1-positive microglial nuclear of inner or outer nuclear also showed the remarkable difference between *WT+BLE* and the other three groups (Figure 5). These results accumulatedly indicated that *Ccr2* knockout could inhibit the microglia activation in the retina, especially under certain pathological conditions, such as chronic blue light-induced retinopathy.

### Blue light-induced activation of the NLRP3 inflammasomes and secretion of IL-1β in microglia in the retina

**Figure 6:**
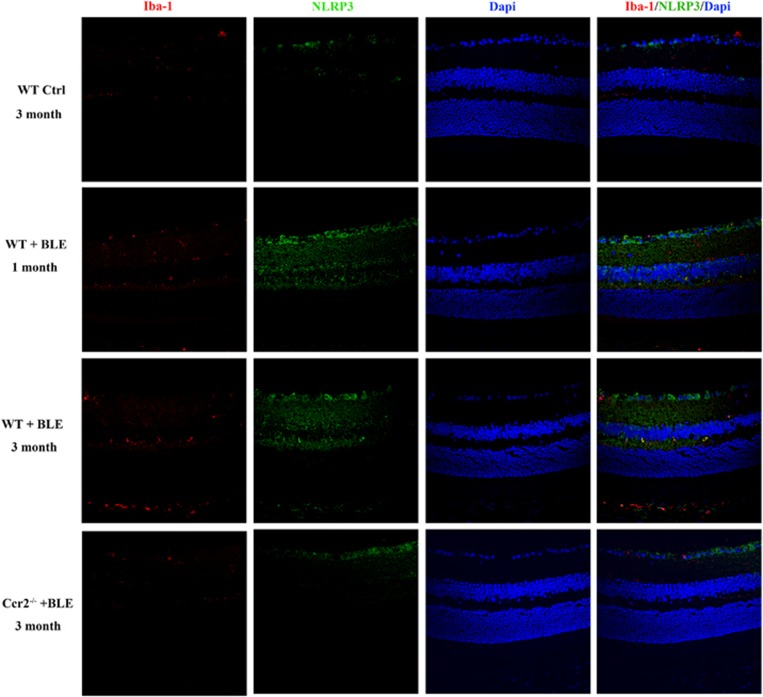
*Ccr2* knockout alleviates the activation of NLRP3 inflammasomes in blue light-induced retinopathy by the impairment of microglial recruitment. NLRP3 immunoactivity (green) was detected co-located with microglia (red) in *WT+BLE* mice after 1 month and 3 months. Cell nuclei (blue) were counterstained with DAPI. The fluorescent photomicrographs indicated that more NLRP3 was found co-localized with Iba1 (arrow) in *WT+BLE* mice retina after 1 month and 3 months compared with that in *WT Ctrl* and *Ccr2*^**−/−**^*+BLE* mice retinas after 3 months

**Figure 7:**
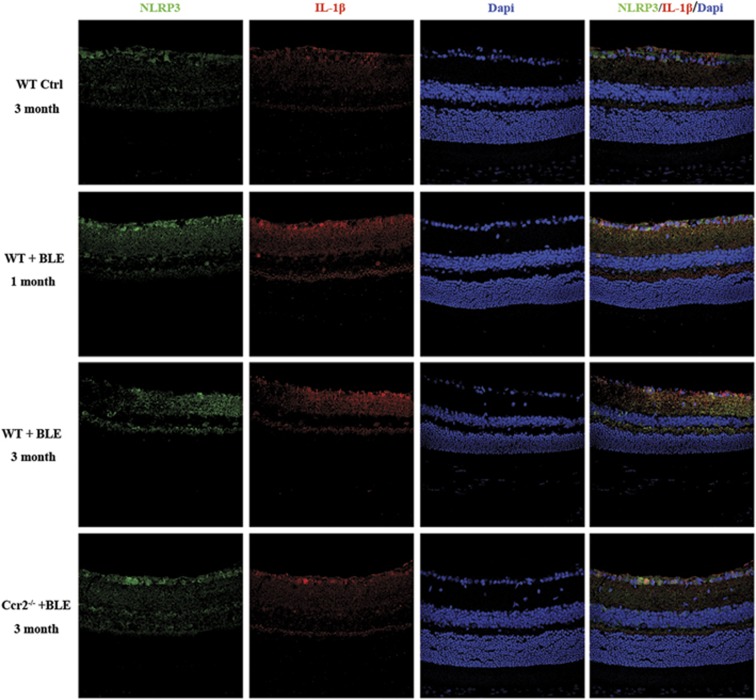
*Ccr2* knockout reduced the secretion of IL-1*β* in blue light-induced retinopathy. IL-1*β* immunoactivity (red) was detected expressed in all groups. Cell nuclei (blue) were counterstained with DAPI. The fluorescent photomicrographs indicated that more IL-1*β* was found co-localized with NLRP3 (green) in *WT+BLE* mice retina after 1 month and 3 months compared with that in *WT Ctrl* and *Ccr2*^**−/−**^*+BLE* mice retinas after 3 months

It has been reported that IL-1*β* is upregulated in the retinas of patient with AMD^34^ and is associated with the death of photoreceptors.^37, 38, 39^ One of the essential pathway of IL-1*β* secretion has been indicated as the activation of NLRP3 in macrophage/microglia in many neurodegenerative disease,^31^ so we next performed immunofluorescence analysis of retinal tissues to determine whether chronic blue light could cause the upregulation of NLRP3 and IL-1*β* in microglia activated in the retina. Figure 6 shows that NLRP3 was consistently elevated in *WT+BLE* mice after 1 month and 3 months. The activated NLRP3 was found co-located with activated microglia, which was also increased by Iba-1 immunofluorescence. Figure 7 shows that with the activation of microglia and NLRP3 in mice exposed to blue light, IL-1*β* was also remarkably secreted and co-located with NLRP3 in the retina of *WT+BLE* mice. Interestingly, we found that NLRP3 and IL-1*β* were also continuously expressed in the *WT Ctrl* mice retina and mainly located at the inner retina (from ganglion cell layer to nuclear cell layer). After BLE, however, more NLRP3 and IL-1*β* were found expressed in the inner retina and outer plexiform layer (Figures 6 and 7). On the other hand, owing to the knockout of *Ccr2* gene and the consequential impairment of microglial recruitment, less NLRP3 was activated and the secretion of its downstream IL-1*β* was also suppressed in *Ccr2*^**−/−**^*+BLE* mice (Figures 6 and 7).

### Ccr2 knockout suppresses the expression of NLRP3, caspase-1, and IL-1β in blue light-induced retinopathy by western blotting analysis

**Figure 8:**
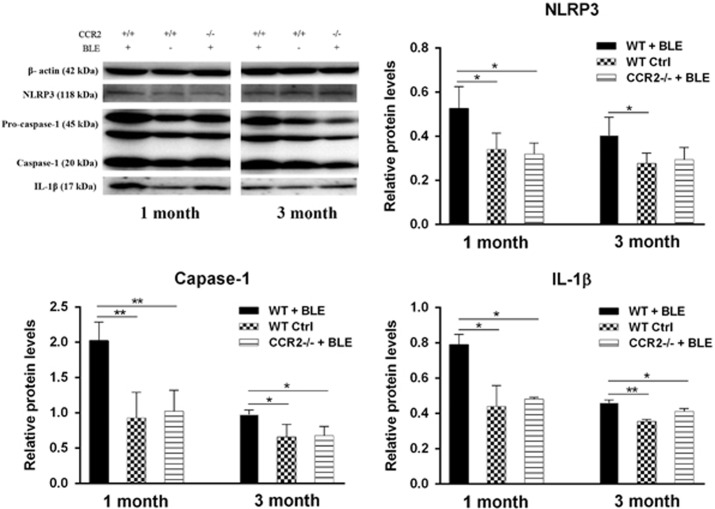
*Ccr2* knockout suppresses the expression NLRP3, caspase-1, and IL-1*β* in blue light-induced retinopathy by western blotting analysis. Expression levels of the indicated proteins were analyzed after 1 month and 3 months by western blotting. Expression is shown relatively to Actin in each group, which was set to 1. *P<0.05, **P<0.01

After 1 month and 3 months of BLE, the transcriptional upregulation of retinal NLRP3 and IL-1*β* paralleled their immunofluorescence evaluation (Figure 8). The relative expression levels of NLRP3 and IL-1*β* in the *WT+BLE* group after 1 month were significantly higher than that in the *WT Ctrl* and *Ccr2*^**−/−**^*+BLE* groups. After 3 months, the relative expression levels of NLRP3 and IL-1*β* in the *WT+BLE* group seemed slightly downregulated compared with the *WT+BLE* group of 1 month, but both were still statistically higher than the *WT Ctrl* and *Ccr2*^**−/−**^*+BLE* groups at 3 months (Figures 8a, b, and d). We also analyzed the expression of activated caspase-1, the increase of which also can be used as an index of NLRP3 inflammasome activation. There were very robust increases in caspase-1 (p20) activity in the *WT+BLE* group after 1 month (*P*=0.005 compared with the *WT Ctrl* group, *P*=0.007 compared with the *Ccr2*^**−/−**^*+BLE* group) and after 3 months (*P*=0.027 compared with the *WT Ctrl* group, *P*=0.033 compared with the *Ccr2*^**−/−**^*+BLE* group) (Figure 7c).

## Discussion

The aim of this study was to analyze the blue light-induced retinal degeneration in mice and whether the impairment of microglia recruitment can alleviate this retinal degeneration. First, by using chronic and low-intensity blue light, we showed the visual dysfunction and obvious death of retinal photoreceptors in *WT+BLE* mice. Second, we demonstrated microglial activation was early induced and persistent up to 3 months with BLE and was accompanied by a co-localization of NLRP3 and IL-1*β* in fluorescein staining and an upregulated protein expression of NLRP3 inflammasome and mature IL-1*β*. Third, we identified that the suppression of the recruitment of microglia by knocking out *Ccr2* gene would result in a pronounced reduction of protein expression of NLRP3, caspase-1, and IL-1*β*, leading to significant structural and functional preservation of the mice retinas. Also, the *Ccr2*^**−/−**^ *Ctrl* group was included to exclude the influence of the *Ccr2* knockout on the results. Collectively, our data demonstrated that activated microglia and the cellular NLRP3 inflammasomes contributed to the severity of BLE-induced retinopathy. Therefore, modulating microglial activation could be a potential treatment strategy to improve photoreceptor survival in chronic light-induced retinopathy.

Microglial activation has been reported to have a dynamic role in the development of retinal degeneration,^29, 30, 40, 41, 42^ serving as a factor amplifying inflammation in pathological states. Several groups have demonstrated that resident microglia in the retina show very limited proliferation, supporting the concept of microglial replenishment by blood-derived myeloid cells.^43, 44, 45^ As a pivotal chemokine for microglia, *Ccr2* has revealed its essential pathogenic role in studies of various inflammatory and degenerative diseases.^27, 46, 47, 48, 49^ In the present study, *Ccr2*^**−/−**^ mice with exposure to blue light had significantly reduced infiltration of microglial cells into the retina. These findings suggest an essential role of *Ccr2* in the pathogenesis of microglia-mediated degenerative disease, which is consistent with previous reports that *Ccr2* is required for efficient recruitment of peripheral monocytes to the pathological tissues in autoimmune uveitis,^46^ retinitis pigmentosa,^48^ autoimmune encephalitis,^47^ and atherosclerosis.^49^

To decipher the mechanism whereby microglia elicits its pathogenic effects in BLE-induced retinopathy, we sought to identify the NLRP3 inflammasone and IL-1*β* that provoke retinal destruction in mice. NLRP3 activation was initially reported to be found in peripheral blood mononuclear cells,^50^ and following the assembly of NLRP3 inflammasome, the secretion of mature IL-1*β* is responsible for cell apoptosis.^37, 39^ In addition, NLRP3-knockout mice have shown a delayed onset of CNS injury. The most widely studied cells related to NLRP3 inflammasome activation in ocular research were RPE cells,^33, 34, 35, 36^ ganglion cell,^51^ and corneal epithelia,^52, 53, 54^ whereas the role of microglia has not been fully stressed.^55^ It is suggested that RPE undergoing significant changes in structure and function may have a central role in AMD pathogenesis. Potentials of AMD-related factors, such as A*β*,^33, 56^ 4-hydroxynonenal,^57^ carboxyethylpyrrole,^34^ and *Alu* RNA transcripts,^35^ have been reported to activate the inflammasome pathway in RPE and further lead to RPE atrophy in AMD progress. However, our work indicated that, during the BLE-induced retinal degeneration, the NLRP3 inflammasome and the following IL-1*β* were mainly expressed in activated microglia. Whereas neither immunofluorescence of the retinal sections nor western blotting analysis of the RPE–choroid tissues indicated an obvious activation of inflammasome and secretion of IL-1*β* (Supplementary Figure S1). Considering the very little quantity of the transcribed protein of NLRP3 inflammasone and IL-1*β* in RPE–choroid complex, here we supposed that microglia might make much more contributions to the BLE-induced retinopathy in mice. Of note, the activation of microglia and the cellular inflammasomes occurred as an earlier step before the structural and functional damage of the mice retinas. After 1 month of BLE, there was no significant reduction of the ONL thickness and ERG responses in WT mice. However, at the same time point, microglia was remarkably activated and accompanied by an increased TUNEL positivity of photoreceptors. Furthermore, the NLRP3, pro-caspase-1, caspase-1, and IL-1*β* were also highly expressed in *WT+BLE* mice after 1 month. To further prolong the exposure duration of blue light, the reduction of ONL thickness and photopic ERG a- and b-wave amplitudes would then be remarkably induced. All the data collectively suggested that the activation of microglia and their cellular inflammsome might be an early alteration in the pathogenesis of retinal degeneration and their continuous activation would finally induce both the functional and structural damage to the retina. Further studies are required to clarify what activators trigger the activation of microglial NLRP3 inflammsome and the interactions between microglia and RPE cells in the more prolonged light-induced retinopathy.

In summary, our study provided strong evidence to support that activated microglia were important contributors to the overall apoptosis of photoreceptors in BLE-induced retinopathy. The activated microglia may induced photoreceptor death by the assembly of intracellular NLRP3 inflammasome and the secretion of IL-1*β* secretion. Our data suggested that therapeutic interventions aimed at preventing the photoreceptor cells loss can be through the modulation of microglia and the cellular NLRP3 inflammasome activation.

## Materials and Methods

### Animals and light exposure

*WT* mice (C57BL/6J) and *Ccr2*^**−/−**^ male mice (004999), aged 8 weeks, were obtained from Jackson Laboratory (Bar Harbor, ME, USA). Animals were housed and maintained at the Laboratory Animal Unit of the Nanjing Medical University. All experimental procedures were approved by the Animal Care and Use Committee of Nanjing Medical University and performed in accordance with the Association for Research in Vision and Ophthalmology Statement for the Use of Animals.

Mice were divided into four groups: *WT Ctrl*, WT mice with BLE (*WT+BLE*), Ccr2^**−/−**^ Ctrl (*Ccr2*^**−/−**^ *Ctrl*), and Ccr2^**−/−**^ mice with BLE (*Ccr2*^**−/−**^*+BLE*) groups. Mice from the Ctrl groups were housed on a 12-h dim light/dark cycle (dim light, ~5 lux). Mice in the BLE groups, as described in our previous work,^58^ were exposed to cool blue light-emitting diodes (transmission peak wavelength: 480 nm, Zhongding Technology Co., Ltd. Shenzhen, China), which were positioned inside the cages on a 12-h light/dark cycle every day for up to 3 months (Supplementary Figure S2). We applied the illuminance intensity of 500 lux as it is measured as the normal room light intensity^59^ and has been reported capable of causing retinopathy.^60, 61^

### Electroretinogram

ERG was recorded on 10 mice in each group using a commercial ERG system (Roland Consult, Brandenburg, Germany). Mice were initially dark adapted overnight and anesthetized intraperitoneally with a cocktail of ketamine (100 mg/kg) and xylazine (10 mg/kg). Corneas were anesthetized with proxymetacaine hydrochloride (0.5% Alcaine; Alcon, Fort Worth, TX, USA) and pupil were dilated with 0.5% tropicamide (Mydrin-P; Santen Pharmaceutical, Osaka, Japan). Body temperature was maintained at 37.5 °C with a heating pad. ERG was measured using a gold wire corneal electrode, a forehead reference electrode, and a ground electrode subcutaneously near the tail. In order to evaluate the rod photoreceptor function (scotopic ERG), five-strobe flash stimuli were presented in a Ganzfeld with flash intensities at 0.0095 cds/m^2^ (−25 dB), 0.095 cds/m^2^ (−15 dB), 0.95 cds/m^2^ (−5 dB), 3.0 cds/m^2^ (0 dB), and 9.49 cds/m^2^ (5 dB). The amplitude of a-wave was measured from baseline to the maximum a-wave trough and b-wave was measured from the trough of a-wave to the peak of b-wave. Both a-wave and b-wave were recorded with a band-pass filtered from 0.3 to 300 Hz.

### Retinal sections

Mice were killed after the ERG recording, and both eyes were surgically harvested. For paraffin-embedded retinal sections: the eyeballs were fixed in 4% paraformaldehyde (PFA; Wuhan Boster Bio-engineering, Wuhan, China) in PBS (0.01 M; pH 7.4) for 24 h at 4 °C. Then the eyeballs were immersed in graded series of ethanol and chloroform for dehydration and embedded in paraffin. Five-micro thickness cross-sections were cut along the vertical meridian using a microtome. Sections containing the optic nerve head were selected for histological and TUNEL analysis. For frozen retinal sections: after fixation with 4% PFA overnight, eyeballs were cut at the ora serrata to remove the lenses. The eyecups (retina, choroid, and sclera) were placed in 30% sucrose solution at 4 °C overnight for cryoprotection. Eyecups were then embedded in O.C.T. Compound (Tissue-Tek, Naperville, IL, USA) and kept at −80 °C. The frozen sections were cut at thicknesses of 8 *μ*m containing the optic nerve head before use.

### Histological analysis

Six paraffin-embedded sections (thickness 5 *μ*m) cut through the optic nerve of each eyecup were stained with H&E. Slides were deparaffinized and underwent a series of xylene and alcohol wash. Images were photographed using a light microscope (Olympus, Tokyo, Japan), and the thickness of the ONL from the optic disc was measured at 200-*μ*m intervals in a masked manner by a single observer (ZH). Data from six sections were averaged for each eye.

### TUNEL staining

TUNEL staining was performed according to the protocols (*In Situ* Cell Death Detection kit; Roche Biochemicals, Mannheim, Germany) and nuclei were stained with 4′, 6-diamidino-2-phenylindole (DAPI, Sigma, St. Louis, MO, USA). Fluorescence images were photographed using a confocal microscope (Olympus 1X81, Olympus, Tokyo, Japan), and positive cells in the ONL at a distance between 240 and 720 *μ*m from the optic disc were counted in the superior area of the retinas. The numbers of TUNEL-positive cells were then averaged for these areas.

### Immunofluorescence

Primary antibodies used in this study were rabbit anti-Iba-1 (1:300; Wako Chemicals, Tokyo, Japan), rat anti-nlrp3 (1:300; R&D Systems, Minneapolis, MN, USA), and rabbit anti-IL-1*β* (1:300; Abcam, Cambridge, MA, USA). Secondary antisera were Alexa Fluor 488 of donkey anti-rat (1:1000; dilution) and Alexa Fluor 594 of donkey anti-rabbit (1:1000 dilution). Immunofluorescence was performed according to the following procedures. Briefly, the frozen sections were first washed with PBS three times (5 mins/time) and blocked with 1% bovine serum albumin (Sigma) solution at room temperature for 1 h. The sections were incubated for 2 h with primary antibodies at 25 °C. After washing with PBST four times (10 mins/time), sections were then incubated with fluorochrome-conjugated secondary antibodies for 1 h. Each of the above steps was followed by four 10-min rinses in PBST. Cell nuclei were counterstained with DAPI. Images were captured using a confocal microscope (Olympus 1X81 microscope). Positive staining of the sections was confirmed by comparing with the negative Ctrls.

### Western blotting

Mice retinas and RPE/choroids were separately sonicated in lysis buffer (NP-40, 50 mM Tris, 150 mM NaCl, 1% Triton X-100) and the total proteins were resolved in 10% Tris-glycine gel. Protein concentration was determined using the Bicinchoninic Acid Protein Assay Kit (Pierce, Rockford, IL, USA). Total protein was electrophoresed on 12% sodium dodecyl sulfate–polyacrylamide gel electrophoresis using Electrophoresis System (Mini-Proten Tetra System, Bio-Rad, Hercules, CA, USA) and then transferred onto a polyvinylidene difluoride membranes (Millipore, Billerica, MA, USA). After blocking with Tris-buffered saline containing 0.1% Tween-20 and 5% skim milk overnight at room temperature, the blot was incubated with primary antibodies NLRP3 (Abcam, ab4207), IL-1*β* (Abcam, ab9722), caspase-1 (Abcam, ab108362), and GAPDH (Bioworld, AP0063, St. Louis, MN, USA) and horseradish peroxidase-conjugated secondary antibodies, respectively. Densitometric analysis of western blotting bands was quantified using the Image J software (National Institutes of Health, Bethesda, MD, USA) and normalized to GAPDH.

### Statistical analyses

One-way ANOVA test followed by Tukey's *post hoc* test was performed to assess the statistical differences between the groups using the SPSS 19.0 software (IBM SPSS, Chicago, IL, USA). All results were expressed as means±S.E.M. A value of *P*<0.05 was considered statistically significant. Figures were obtained by the GraphPad Prism 4 software (GraphPad Software Inc., San Diego, CA, USA).

## Abbreviations

AMD: age-related macular degeneration
CCR2: C-C motif chemokine receptor-2
CCR2^−/−^: CCR2 knockout
NLRP3: Nod-like receptor family pyrin domain containing 3
ERG: electroretinogram
CNS: central nervous system
RPE: retinal pigment epithelium
WT: wild-type
BLE: blue light exposure
Ctrl: control
ONL: outer nuclear layer
TUNEL: terminal deoxynucleotidyl transferase (TdT)-mediated dUTP nick-end labeling
Iba-1: ionized calcium-binding adaptor molecule 1
IL-1*β*: interleukin-1 beta
