# Supplementary material for: Knockout of Ccr2 alleviates photoreceptor cell death in rodent retina exposed to chronic blue light
Source: Cell Death Dis. 2016 Nov 10;7(11):e2468–. doi: 10.1038/cddis.2016.363 (PMC5260896; doi:10.1038/cddis.2016.363)
Supplement: Supplementary Table S1 [file cddis2016363x1.doc]

| Supplementary table 1. Quantification of activated microglia in retina. | | | | | | |
| --- | --- | --- | --- | --- | --- | --- |
|  | 1 month | | | 3 month | | |
|  | Control | BLE | *p*^#^ | Control | BLE | *p*^#^ |
| Wild-type | 11.66 ± 3.14 | 32.00 ± 9.89 | 0.000 | 12.00 ± 2.24 | 35.50 ± 3.28 | 0.000 |
| Ccr2^-/-^ | 6.75 ± 3.59 | 7.20 ± 4.20 | 0.970 | 11.60 ± 5.86 | 10.00 ± 1.41 | 1.000 |
| *p*^##^ value | 0.006 | 0.000 | - | 0.309 | 0.000 | - |
| BLE, blue light exposure. ^#^Comparison between control and BLE groups for wild-type or *Ccr2^-/-^* mice. ^##^Comparison between wild-type and *Ccr2^-/-^*mice receiving the same treatment (control or BLE). | | | | | | |
