# Supplementary figures and images for: Knockout of Ccr2 alleviates photoreceptor cell death in rodent retina exposed to chronic blue light
Source: Cell Death Dis. 2016 Nov 10;7(11):e2468–. doi: 10.1038/cddis.2016.363 (PMC5260896; doi:10.1038/cddis.2016.363)

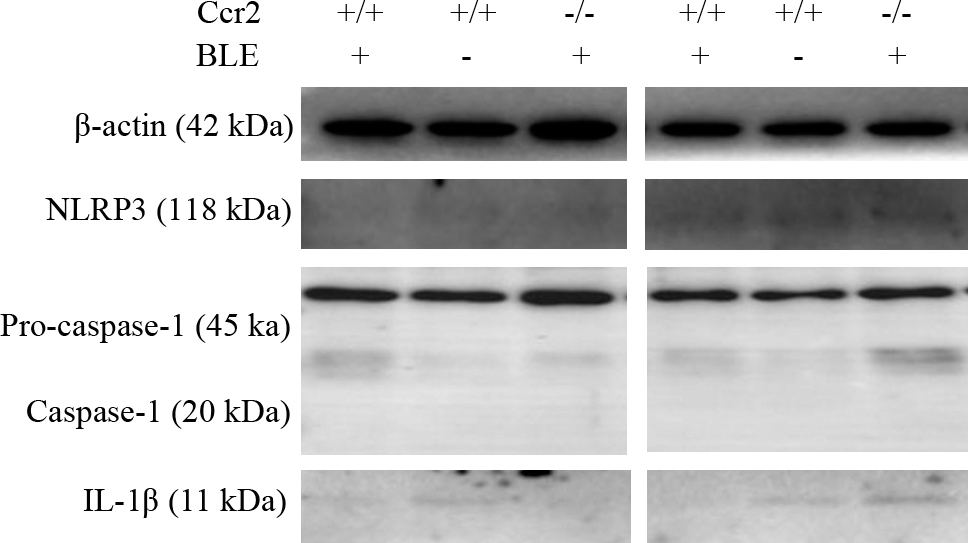

Supplement: Supplementary Figure S1 [file cddis2016363x2.tif]

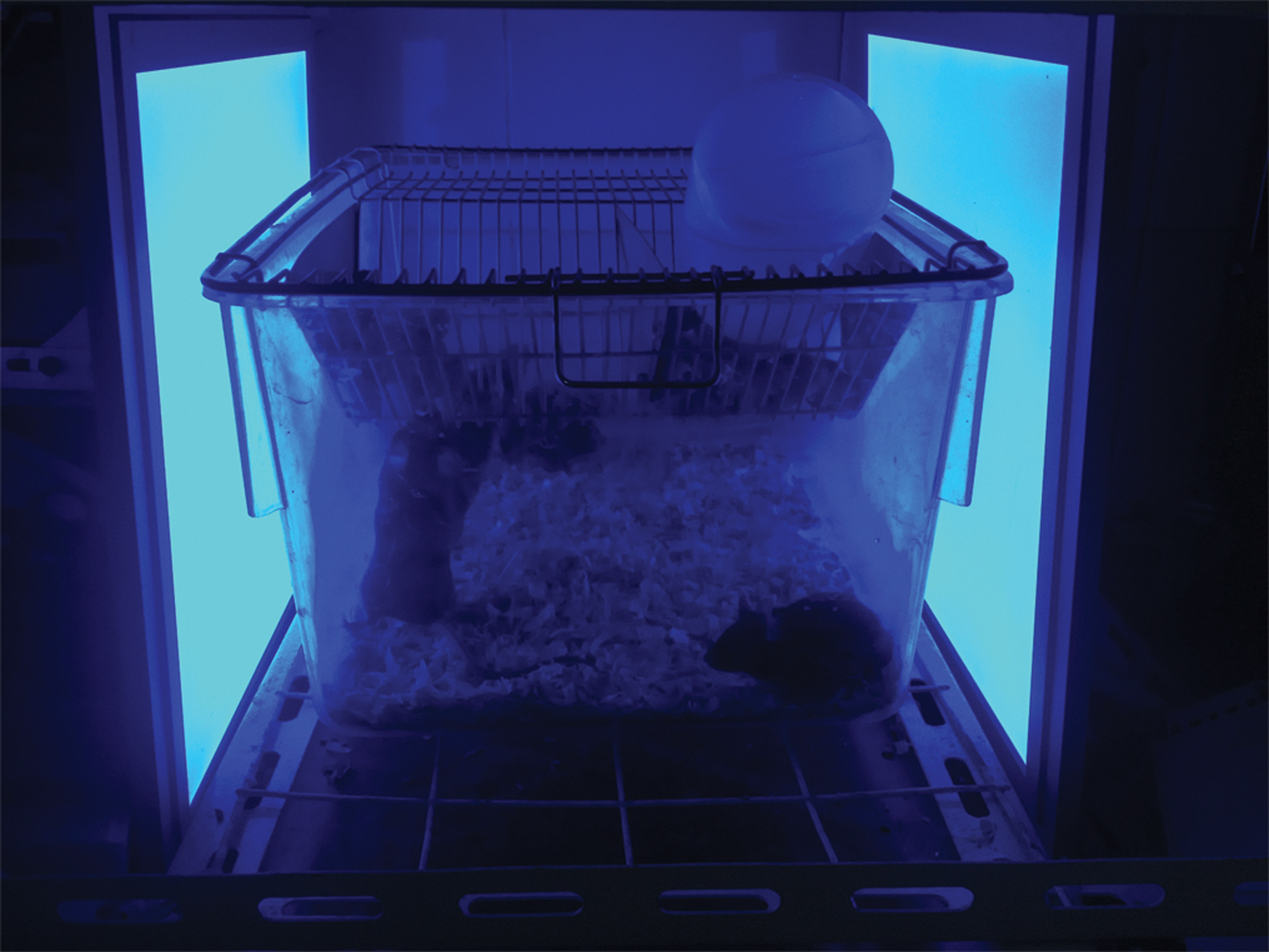

Supplement: Supplementary Figure S2 [file cddis2016363x3.tif]
